# Calcium Phosphate Particles Coated with Humic Substances: A Potential Plant Biostimulant from Circular Economy

**DOI:** 10.3390/molecules26092810

**Published:** 2021-05-10

**Authors:** Alessio Adamiano, Guido Fellet, Marco Vuerich, Dora Scarpin, Francesca Carella, Clara Piccirillo, Jong-Rok Jeon, Alessia Pizzutti, Luca Marchiol, Michele Iafisco

**Affiliations:** 1 Institute of Science and Technology for Ceramics (ISTEC), National Research Council (CNR), Via Granarolo 64, 48018 Faenza, Italy; francesca.carella@istec.cnr.it (F.C.); Michele.iafisco@istec.cnr.it (M.I.); 2 Department of AgriFood, Animal and Environmental Sciences, University of Udine, via delle Scienze 206, 33100 Udine, Italy; guido.fellet@uniud.it (G.F.); vuerich.marco@spes.uniud.it (M.V.); scarpin.dora@spes.uniud.it (D.S.); pizzutti.alessia@spes.uniud.it (A.P.); luca.marchiol@uniud.it (L.M.); 3 Institute of Nanotechnology (NANOTEC), National Research Council (CNR), Campus Ecoteckne, Via Monteroni, 73100 Lecce, Italy; clara.piccirillo@nanotec.cnr.it; 4 Department of Agricultural Chemistry, Food Science & Technology, IALS, Gyeongsang National University, Jinju 52828, Korea; jrjeon@gnu.ac.kr; 5 Department of Life Sciences, University of Trieste, Via Licio Giorgieri 10, 34127 Trieste, Italy

**Keywords:** calcium phosphate, humic substances, plant biostimulants, circular economy, phosphorous, nutrients uptake

## Abstract

Nowadays, the use of biostimulants to reduce agrochemical input is a major trend in agriculture. In this work, we report on calcium phosphate particles (CaP) recovered from the circular economy, combined with natural humic substances (HSs), to produce a plant biostimulant. CaPs were obtained by the thermal treatment of *Salmo salar* bones and were subsequently functionalized with HSs by soaking in a HS water solution. The obtained materials were characterized, showing that the functionalization with HS did not sort any effect on the bulk physicochemical properties of CaP, with the exception of the surface charge that was found to get more negative. Finally, the effect of the materials on nutrient uptake and translocation in the early stages of development (up to 20 days) of two model species of interest for horticulture, *Valerianella locusta* and *Diplotaxis tenuifolia,* was assessed. Both species exhibited a similar tendency to accumulate Ca and P in hypogeal tissues, but showed different reactions to the treatments in terms of translocation to the leaves. CaP and CaP–HS treatments lead to an increase of P accumulation in the leaves of *D. tenuifolia*, while the treatment with HS was found to increase only the concentration of Ca in *V. locusta* leaves. A low biostimulating effect on both plants’ growth was observed, and was mainly scribed to the low concentration of HS in the tested materials. In the end, the obtained material showed promising results in virtue of its potential to elicit phosphorous uptake and foliar translocation by plants.

## 1. Introduction

Intensive farming has been extensively adopted in recent years to cope with the increasing global food demand [1]. Unfortunately, this practice often fails sustainability principles as it requires the use of massive quantities of pesticides and nutrients involving the depletion of non-renewable resources. In this context, the use of biostimulants for increasing plant nutrient use efficiency (NUE) have been proposed several times to reduce the chemical inputs of intensive farming while maintaining high productivity levels [2].

Among the various biostimulants, humic substances (HS) are among the most studied in virtue of (i) their ability to stimulate plant growth and increase nutrient uptake and hormone production; (ii) their natural occurrence in soils; and (iii) their positive interaction with soil bacterial communities [3,4]. As an example, Purwanto et al. studied the effects of different combinations of HS extracted from composted manure and P_2_O_5_ on different corn cultivars [5], reporting a substantial growth in the crop yields due to an increase in the crop root dimensions—that in turn boosted the P uptake—to a significant improvement of the physiological performance of plants. HSs were also found to elicit physiological processes of plants, promoting abiotic stress resistance, in particular salt tolerance and drought stress, in several plants such as *Capsicum annuum*, *Oryza sativa,* and *Phaseolus vulgaris* [6,7,8,9]. Moreover, it has been proved that HSs have the ability to interact with calcium phosphates [10] and increase P bioavailability in the soil, often present in the form of insoluble complexes [6].

In a previous work, some of the authors have functionalized synthetic hydroxyapatite nanoparticles, which were already proposed for agronomic applications [11], with soil friendly HS to produce a nanocarrier that could efficiently deliver both nutrients and biostimulants toward plants [12]. The results obtained on corn plants showed that the co-release of P and HS from the nanoparticles leads to a boost of crop biomass growth and of abiotic stress resistance. In another work, we showed that calcium phosphate particles (CaP) extracted from fish bones at different temperatures (i.e., in the 300–900 °C range) have the ability to increase the germination rate of *Lepidium sativum* seeds, and to boost the growth of *Zea mays* coleoptiles and plants [13].

Thus, in an attempt to produce a biostimulant from circular economy with potential agronomic applications, here we report on the production of CaP obtained from the thermal treatment of salmon (*Salmo salar*) bones and on their engineering with natural HS. The rationale behind this study is that HS could be combined with CaP to obtain a biostimulant with improved performances. The obtained materials were applied on *Diplotaxis tenuifolia* and *Valerianella locusta* that were chosen as test species in virtue of their wide use in horticulture. Attention was paid to investigate the different effects on the early growth (up to 20 days), nutrient uptake, and elements translocation from the roots to the leaves, and highlight species-specific responses by the two plants.

## 2. Results

### 2.1. Materials Characterization

**Figure 1:**
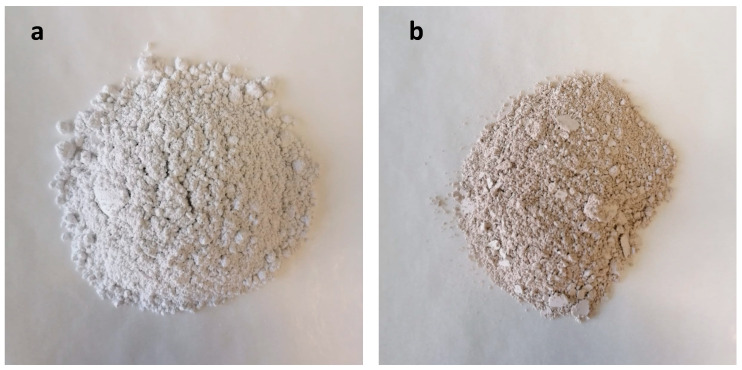
Pictures of the calcium phosphate before (**a**) and after soaking in a humic acid solution at 0.1 g mL^−1^ (**b**).

Pictures of the materials obtained from the calcination of salmon bones and of those derived from its functionalization with HS are reported in Figure 1. The sample without HS treatment is named CaP hereafter, while that obtained by soaking CaP in a water solution of HS at 0.1 g L^−1^ is named CaP–HS. The functionalization with HS changed the color of the materials from the white of CaP to the brownish/greyish of CaP–HS.

**Figure 2:**
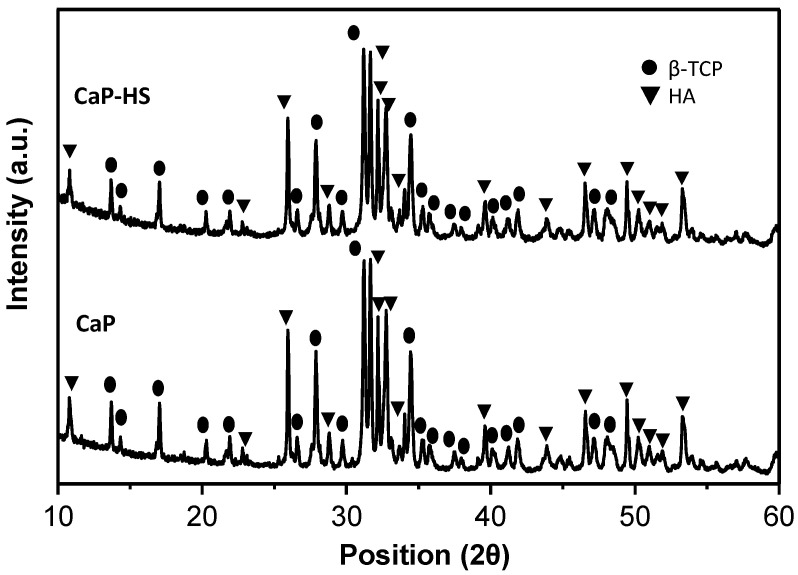
XRD patterns of bare CaP and CaP functionalized with HS (CaP–HS).

Samples were analyzed by XRD, and the collected spectra are reported in Figure 2. The XRD patterns indicate that both the samples consist of a bi-phasic mixture of beta-tricalcium phosphate (β-TCP, β-Ca_3_(PO_4_)_2_) and hydroxyapatite (HA, Ca_10_(PO_4_)_6_)). In more detail, the spectra are featured by the occurrence of peaks typical of HA, located at 2θ values of 25.9°, 31.7°, 32.9°, 34.0°, 46.7°, and 49.5° corresponding to the lattice planes with Miller indexes (0 0 2), (2 1 1), (3 0 0), (2 0 2), (2 2 2), and (2 1 3), respectively, and of peaks typical of β-TCP located at 2θ values of 13.6°, 17.0°, 27.8°, 31.0°, and 34.3°, corresponding to the lattice planes (1 0 4), (1 1 0), (2 1 4), (0 2 10), (2 2 0), respectively. Finally, all of the spectra are characterized by the occurrences of sharp and resolved peaks with no difference between bare CaP and CaP–HS.

The crystallinity indexes (CI) calculated according to Equation (1) are displayed in Table 1, together with the phase compositions calculated by Rietveld refinement, the analysis of the main element by ICP–OES and the amount of HS determined by TGA. Both the materials are characterized by the occurrence of β-TCP and by HA with a weight ratio close to 60:40, respectively, with no difference between bare CaP and CaP–HS. The CI shows that the samples are all highly crystalline, as typically reported in the literature for calcium phosphates obtained by treating fish bones at temperatures around 800 °C, and that the HS functionalization did not have any effect on the crystallinity [13,14]. The ICP results show that samples have a similar chemical composition with no statistically significant difference among them for Ca, P, and Mg content (student-*t* test, *p* < 0.05). Finally, CaP has a slightly higher content of K and Na with respect to CaP–HS.

**Figure 3:**
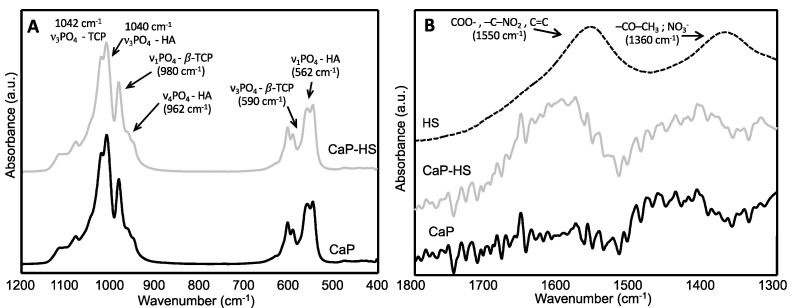
FT-IR spectra of CaP, CaP–HS and HS. The range of the spectra where the main IR bands of HA and β-TCP appear (1200–400 cm^−1^) is reported on the left (**A**), while a magnification of the FT-IR spectra in the region where the typical IR bands of HS (1800–1300 cm^−1^) occur is reported on the right (**B**).

The ATR spectra of the materials are reported in Figure 3 and confirmed the occurrence of β-TCP and HA in all the samples. The main IR bands of these phases, corresponding to the triply degenerated asymmetric stretching vibration mode (ν3) of the phosphate tetrahedron, are really close, 1040 and 1042 cm^−1^ for HA and β-TCP, respectively, and are superimposed. However, the typical β-TCP bands are visible at 980 and 945 cm^−1^, corresponding to the stretching mode (ν1) of PO_4_, and at 590 and 550 cm^−1^, corresponding to the triply degenerated bending mode (ν4) of PO_4_ [15]. Characteristics HA bands corresponding to the triply degenerated bending mode (ν4) and to the asymmetric stretching mode (ν1) of PO_4_ are also visible at 962 and 562 cm^−1^, respectively [16,17]. On the other hand, the spectrum of pure humic acid is characterized by signals ascribable to COO^-^, –C–NO_2_, and C=C groups (1550 cm^−1^), and to –CO–CH_3_ and possibly nitrate groups (1360 cm^−1^) [18]. These signals are absent in the spectrum of CaP, but the signal at 1550 cm^−1^ is present in that of CaP–HS, confirming the presence of HS as already detected by TGA.

The ζ-potential of the particles determined by DLS and reported in Table 1 was found to be more negative for the CaP coated with HS respect to the bare one. Finally, the engineering of the particles surface did not sort any effect on the specific surface area of CaP–HS (8.50 m^2^ g^−1^) that was very close to that of CaP (8.53 m^2^ g^−1^).

**Figure 4:**
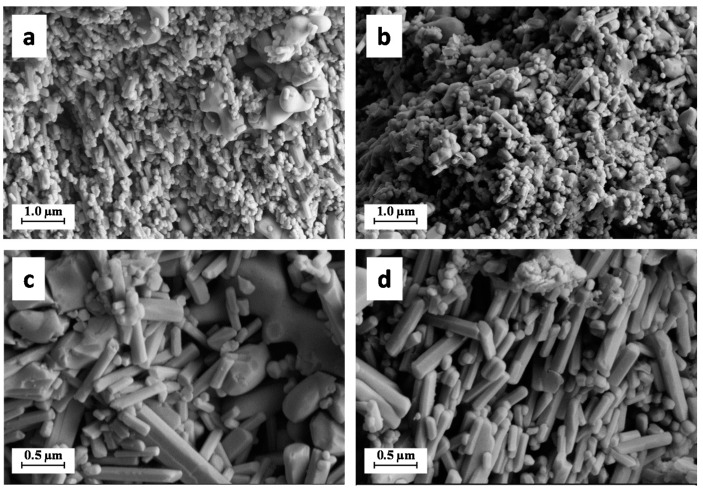
SEM pictures of CaP (**a**,**c**) and CaP–HS (**b**,**d**) at 50,000× magnification on the top (**a**,**b**) and 100,000× magnification on the bottom (**c**,**d**).

Micrographs of the materials at different magnifications recorded by SEM are reported in Figure 4. All the samples are featured by the occurrence of two kind of particles with a different morphology: (i) coarser and flattened particles with round shape and smooth edges, with a size in the range 1.0–2.0 μm; and (ii) elongated rod-like particles with a minor axis in the size range 0.05–0.10 μm and a major axis with size in the range 0.1–1.0 μm. The first morphology can be ascribed to β-TCP crystals, while the second one can be ascribed to HA [19]. No difference can be noticed between bare CaP and CaP coated with HS.

### 2.2. Observations on Plant Species

#### 2.2.1. Germination and Seedlings Development

A two-way ANOVA was run within the species *D. Diplotaxis* and *V. tenuifolia* to highlight the effects of treatments (Tables S1–S4). Interaction effects represent the combined effects of experimental factors on the dependent parameter.

The recorded observations show that the experimental treatments did not influence seeds germination percentage. Although non-treated seeds had the lowest percentage of germination for both species, data variability has hidden statistical evidence of response to the treatments (Tables S3 and S4), which were not significant by ANOVA (*p* = 0.7834 and *p* = 0.2106, respectively for *D. tenuifolia* and *V. locusta*).

Regarding the root length, the species’ response was different, this difference being statistically significant (*p* < 0.01) for *V. locusta* (Table S2) and not significant for *D. tenuifolia* respectively. In the case of *D. tenuifolia*, all treatments resulted in a reduction in the root system’s development (Table 2), with seedlings treated with CaP having −12.8% in root length if compared to control plants. A similar effect was recorded for CaP–HS (−13.3%). On the contrary, in *V. locusta* seedlings the root system’s development was significantly stimulated by HS and the combination of HS and CaP by 6% and 20% more compared to control, respectively (Table 3).

The accumulation of dry matter in the root tissues of *D. tenuifolia* responded significantly to treatments (Table S1). In details, the presence of CaP alone or in combination with HS stimulated a significant increase in the dry weight of the roots of +50.1% and +23.6% compared with control, respectively (Table 2). On the other hand, a negative trend for HS, although not statistically significant, was recorded for both the treatments with HS, i.e., HS alone and in combination with CaP (Table 2).

In the case of *V. locusta*, treatments did not affect significantly roots dry weight; however, a different response could be observed with respect to *D. tenuifolia*, since the stimulating effect of CaP was practically nullified (Table 3). The dry matter accumulation was the highest with CaP–HS treatment (7.73 mg plant^−1^), equal to +24% with respect to the control.

The root specific weight was calculated by combining roots’ cumulative length and dry weight. In both the species, this variable was not affected by the imposed treatments (Tables S3 and S4).

Regarding the dry matter accumulation in the aerial part of plantlets, *D. tenuifolia* was affected by the interaction of CaP and HS (*p* < 0.05) (Table S1). An increase (+19%) was observed in the presence of CaP alone, whereas for CaP-HS, the shoot dry weight was similar to the control (Table 2). A more pronounced increase in DM shoot accumulation was recorded in *V. locusta* (Table 3). In this case, the treatment responses were statistically significant for HS (*p* = 0.002 *, Table S2). The effect of CaP was negligible, whereas HS stimulated the shoot biomass production (+19.8% and +13.5% compared to control, respectively) (Table 2).

The total DW per plant was calculated by adding the dry weight of plantlet fractions (Tables S3 and S4). According to the measured data, the species’ treatment response was not significant for both species.

In the experimental conditions, the shoots’ energetic status represented by ATP concentration was not influenced by the treatments. No statistically significant differences were observed in both species (Table 2 and Table 3).

#### 2.2.2. Element Concentration in Seedling Roots and Shoot

Considering the two species separately, the effect of treatments on nutrients concentration was noticeably different, both in roots and leaves, but similarities were highlighted especially for P and Ca concentrations, whose high correlation in the global data set is certified by Figure S1.

**Figure 5:**
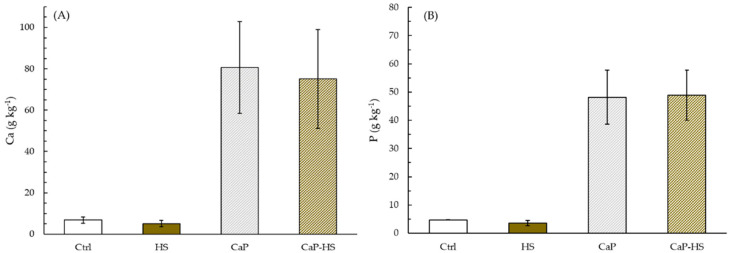
Concentration of Ca (**A**) and P (**B**) in root of *Diplotaxis tenuifolia*. Data are mean ± standard deviation (n = 4). When the interaction between experimental factors (CaP × HS) was significant at ANOVA, different letters were used to indicate statistically significant differences between treatments at Tukey’s post-hoc test (*p* ≤ 0.05).

In *D. tenuifolia,* the effect of CaP on the Ca level in the roots was significant (*p* < 0.001), with an increase in its concentration regardless of the presence of HS (Figure 5A). A similar result was found for the concentration of P in roots, with the same degree of significance (Figure 5B).

The Mg level in root was significantly modulated by CaP and HS factors (*p* < 0.001, *p* < 0.05), showing a decreasing trend in concentration in the presence of HS if compared to control or CaP alone, which instead slightly increased Mg content (Figure S2). The level of K in roots was affected mainly by HS treatment (*p* < 0.01), while the interaction effect between factors was low (*p* = 0.027): the presence of HS seemed to reduce K content in roots, with a strong depression particularly given by HS alone compared to the control (Figure S2).

**Figure 6:**
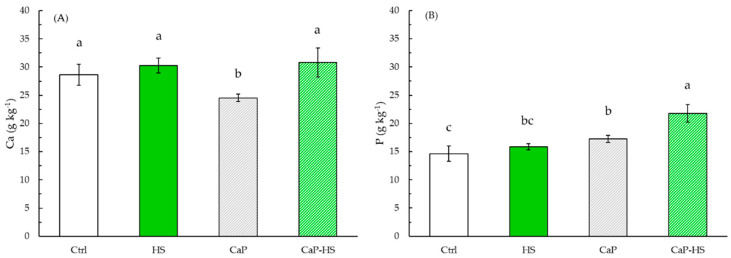
Concentration of Ca (**A**) and P (**B**) in leaves of *Diplotaxis tenuifolia*. Data are mean ± standard deviation (n = 4). Different letters indicate statistically significant differences between means at Tukey’s post-hoc test (*p* ≤ 0.05).

Data obtained from leaf analysis concerning P level showed a strong statistical significance for CaP and HS treatments (*p* < 0.001), and a lower significance value for their interaction (*p* < 0.05) (Figure 6B). However, the level of P increased in plants treated with CaP and CaP–HS with an upwards trend, where the major effect was given by CaP–HS.

Regarding Ca concentration, CaP and HS factors and their interaction were all significant (*p* < 0.05) (Figure 6A). Differently from P nutrient, Ca level was reduced in leaves by the application of CaP, whereas the application of both HS and CaP–HS resulted in the same Ca content of the control.

A statistical significance was also shown for HS factor (*p* < 0.05) and its interaction with CaP (*p* < 0.01) regarding K concentration in leaves: when CaP was functionalized with HS, a K-increasing effect was recorded with respect to both CaP and HS alone (Figure S3).

Considering the effect on Mg, the treatment with CaP–HS was weakly significant (*p* = 0.037). The only difference respect to the control was a slight decrease recorded for CaP treatment (Figure S3).

**Figure 7:**
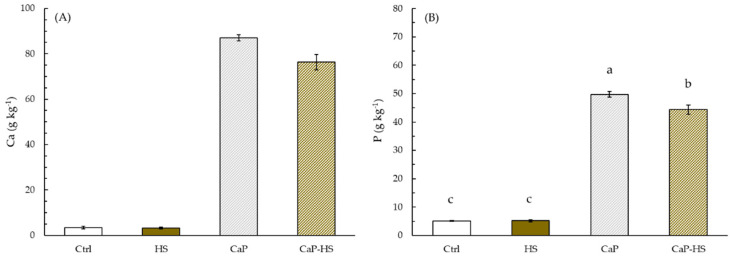
Concentration of Ca (**A**) and P (**B**) in roots of *Valerianella locusta*. Data are mean ± standard deviation (n = 4). When the interaction between experimental factors (CaP × HS) was significant at ANOVA, different letters were used to indicate statistically significant differences between treatments at Tukey’s post-hoc test (*p* ≤ 0.05).

The response of ANOVA on data of *V. locusta* roots concerning P content showed a strong statistical significance for CaP factor (*p* < 0.001), while HS and CaP–HS factors were less significant (*p* < 0.05). While HS applied alone has given no effect on P concentration in roots compared to the control, CaP and CaP–HS showed a noticeable increase in its level, with the greatest effect being expressed by CaP (Figure 7B).

A similar trend was also observed on Ca concentration in *V. locusta* roots (Figure 7A), but in this case only CaP factor was statistically significant (*p* < 0.001).

The tested treatments did not sort any effect on the Mg concentration in roots (data not shown), while a moderate significance (*p* < 0.01) for HS was observed from the data relating to K (Figure S4). In fact, pure HS application compared to the control showed a growing effect on K level, while when applied with CaP microparticles, the trend was the opposite.

**Figure 8:**
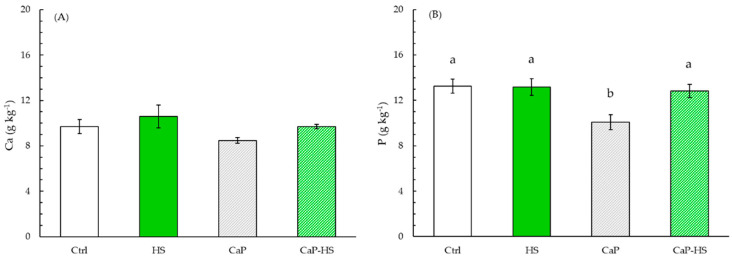
Concentration of Ca (**A**) and P (**B**) in leaves of *Valerianella locusta*. Data are mean ± standard deviation (n = 4). When the interaction between experimental factors (CaP × HS) was significant at ANOVA, different letters were used to indicate statistically significant differences between treatments at Tukey’s post-hoc test (*p* ≤ 0.05).

Considering the effects on *V. locusta* leaves, also in this case we could observe a similarity between treatment’s effects on P and Ca concentrations: in both of them, CaP and HS effects were statistically significant, but in the case of P, the statistical value was stronger for CaP (*p* < 0.001). Moreover, also the interaction between factors was significant for P (*p* < 0.01). In particular, when CaP was applied alone, there was a strong reduction in the concentration of P, while neither HS nor CaP–HS changed P content in leaves compared to the control (Figure 8B).

As for Ca level, instead, CaP and HS were equally significant (*p* < 0.01) (Figure 8A). CaP addition to nutritive substrate decreased Ca content in leaves with respect to the other treatments. On the contrary, the presence of HS, both alone and in CaP–HS, showed an increase in Ca concentration if compared with the two treatments without HS.

Finally, data regarding K and Mg concentration in leaves showed no statistical significance for none of the factors (Figure S5).

## 3. Discussion

The CaP sample has been thermally extracted from salmon bones at 800 °C to be then functionalized by soaking in a water solution of HS.

As largely known in the literature, the thermal treatment of fish bones can lead to the formation of HA and β-TCP at different ratio depending on the fish species and on the temperature of treatment (usually, the higher is the temperature the larger the amount of β-TCP) [20,21]. For instance, some of the authors have previously reported that the thermal extraction of calcium phosphates from *S. aurita* bones at 900 °C leads to the formation of HA as the principal phase, and of just minor quantities of β-TCP (c.a. 5.0 wt.%) [13] differently from other fish species, such as salmon [14] and mackerel (c.a. 40.0 wt.% of β-TCP) [22]. The materials obtained from *S. aurita* are composed mainly by HA in the form of rod-like particles almost identical to the ones detected in this work for the materials extracted from *S. salar*, but do not feature flattened and larger particles ascribable to β-TCP.

The functionalization with HS did not have any effect on the main physicochemical properties of CaP. The only parameter that was found to change among the samples is, as expected, the presence of HS that was confirmed by ATR and quantified by TGA, and the ζ-potential of the particles. More in detail, the ζ-potential of bare CaP was found to be close to that of synthetic HA [11,23], while it got more negative after the functionalization with HS. This increase in the net negative charge of the particles is due to the occurrence of HS on the surface of the CaP, and in particular to the oxygenated functional groups of natural HS getting a partial negative charge in water at neutral pH [24,25]. These data suggest that when HS is added to preformed CaP, the interaction is only superficial and HS molecules stay on the surface of the particles without penetrating the material, with no effect on its bulk structure. This is in line with what already reported in the literature for synthetic HA nanoparticles functionalized with both natural and synthetic humic substances [11,26].

HS was used in virtue of its biostimulant activity on plants and its ability to increase the water solubility of calcium phosphate particles. In more detail, HSs are complex and recalcitrant organic polymers naturally occurring in soils, having the ability to stimulate plants metabolism through genes activation, empowering their resistance to abiotic stress and increasing their germination and growth [25,27].

The aim of this work was to verify whether the coupling of HS with CaP could be useful, not only to promote the germination and development of seedlings through biostimulation, but also to facilitate the intake of nutrients in a synergistic way. In more detail, the main focus was assessing the beneficial effect on P uptake, given its limited bioavailability in mineral calcium phosphate in neutral and alkaline substrates.

What has been observed is that the response of the two chosen species was different, but, considering the effects on nutrients intake, both have shown some similarities on P and Ca concentrations, particularly in hypogeal tissues. Specifically, even if CaP has a low solubility, here it was found to be the main factor that affected P and Ca uptake in roots. On the contrary, it was observed a null effect given by HS for *D. tenuifolia*, and a possible worsening in P level in the case of *V. locusta*, when microparticles were coated with HS. This fact could be explained with a possible trade-off between the increase in CaP dissolution due to a more acidic surface and a limited contact between phosphorus and water molecules caused by HS functionalization, as already highlighted in a previous work with synthetic HA nanoparticles [11].

Considering the effect of treatments on shoots, the most interesting finding was related to P foliar content in *D. tenuifolia*: the application of CaP increased the level of P, which was further enhanced by CaP-HS. This shows the possibility of positive interaction between CaP and HS in increasing P availability, through nutrients chelation and gradual release by HS. In agreement with these results, other reports show the positive effect of humic acid applications in phosphorus fertilization [28,29] and, even more noteworthy, in calcareous soils [30,31,32].

On the contrary, in *V. locusta* Ca content in leaves was enhanced only by HS, regardless of CaP functionalization. In this case, despite an increased root absorption of this element in presence of CaP alone or CaP–HS, Ca translocation to the epigeous portion was significantly stimulated by HS, suggesting that HS could favor the xylematic transport of this macronutrient by a hormone-like activity [3,33].

At the foliar level, a similar trend was observed for Ca in the leaves of *D. tenuifolia* and Ca and P in those of *V. locusta* seedlings treated with CaP, whose concentrations were found to decrease with respect to the control. To explain these results, we hypothesized that the amount of nutrient in the medium was already sufficient to sustain plantlet metabolism and, therefore, the addition of further Ca and P through the microparticles was irrelevant or even unfavorable. In this case, the HS on the surface of CaP probably performed a restorative function through chelation, releasing the nutrients more gradually and allowing Ca and P regular translocation to the leaves; this allowed the achievement of the same saturation level already observed in the control thesis.

The fact that in some cases HS have not shown the expected biostimulating effect can be explained considering the great variability of both the experimental conditions (low concentration on the particles) and of the characteristics of the humic substances used in similar research works. For instance, Nikbakht et al. (2008) highlighted contrasting results on the effect of HS on plant nutrition, and argued that this might be partially related to different soil or growing media, origin and concentration of HS and the species treated [34]. In this light, we found a strong significance in variance of the nutrient content in foliar portion described by the species factor; in particular, it has to be underlined that *D. tenuifolia* seems to constitutively accumulate greater amount of Ca in leaf compared to *V. locusta* (approximately 30% more), confirming a feature that is inherent to other species of *Brassicaceae* [35], although no difference was observed among species at the root level.

On the contrary, in *V. locusta* this feature was not observed, despite both Ca transport to shoot and growth stimulation (described as shoot dry weight and root length increase) were dependent on HS application alone or in combination with CaP. Such evidence suggests that this species probably adopts different absorption and translocation mechanisms.

## 4. Materials and Methods

### 4.1. CaP-HS Production and Characterization

#### 4.1.1. CaP Extraction from Fish Bones

Salmon (Salmo salar) fish bones resulting from the filleting were collected from a local shop (Italy). Before any treatment, fish bones were separated from offal and heads, scraped and soaked in hot water (80 °C) up to 2 h to remove organic tissues, placed on paper towel, and then dried in an oven at 50 °C overnight. The bones were then placed in an open furnace and heated in air by a 100 °C h^−1^ thermal ramp followed by a one-hour isotherm at the temperature of 800 °C. After that, the resulting material was cooled at room temperature and then placed in a mortar to be grinded. The resulting powder was finally passed through a 270 mesh sieve before being characterized and used.

#### 4.1.2. CaP Coating with HS

The coating of CaP extracted from salmon bones with HS (humic acids, Mycsa, AG, Brownsville, TX, USA) was performed as already reported by Yoon et al. [11] with slight modification. Briefly, HS was dissolved in autoclaved distilled water at 0.10 g mL^−1^. The solution was centrifuged at 13,000 rpm for 10 min to remove water-insoluble HS; after, 1 g of CaP was added to 10 mL of the resulting solution to be vigorously vortexed and then placed under gentle agitation in an incubator for 24 h at room temperature. To collect HS-coated CaP, the solution was centrifuged at 13,000 rpm for 10 min and the resulting pellet was rinsed with distilled water. Finally, the materials were recovered by centrifugation and dried overnight at 50 °C.

#### 4.1.3. Samples Characterization

The morphology of the samples was analyzed by scanning electron microscopy with a field-emission microscope (FEG–SEM, ΣIGMA, ZEISS NTS GmBH, Oberkochen, Germany). The samples were powdered and deposited on carbon tape mounted on an aluminum SEM stub and sputter-coated (Polaron E5100, Polaron Equipment, Watford, Hertfordshire, UK) with 10 nm of gold for electrical conductance. Accelerating voltages in the 3 to 10 keV range were used to observe the samples in the secondary electron imaging mode.

Fourier transform infrared spectroscopy analyses in the attenuated total reflection mode (FTIR–ATR) were carried out using a Nicolet iS5 spectrometer (Thermo Fisher Scientific Inc., Waltham, MA, USA) with a resolution of 1 cm^−1^ by accumulation of 16 scans covering the 4000 to 400 cm^−1^ range, using a diamond ATR accessory model iD7.

Thermal Gravimetric Analysis (TGA) were performed using STA 449 Jupiter (Netzsch GmbH, Selb, Germany) apparatus. About 10 mg of powdered samples were heated from room temperature to 1100 °C under air flow with a heating rate of 10 °C min^−1^ in an alumina crucible. The amount of HS on CaP–HS was determined as the weight loss occurring between 550 °C and 950 °C.

Dynamic Light Scattering (DLS) analyses were performed by using a Zetasizer Nano instrument ZSP (Malvern Instruments, Worcestershire, UK). For the analysis, samples were dispersed in water at a concentration of 0.5 mg mL^−1^ at pH 7.0. ζ-potentials were quantified by laser Doppler velocimetry as the electrophoretic mobility at 25 °C using a disposable electrophoretic cell (DTS1061, Malvern Ltd., Worcestershire, UK) of three separate measurements (maximum 100 runs each). Hydroxyapatite refractive index (1.63), water refractive index (1.33), and viscosity (1 cps) were used as working parameters for the samples and the solvent, respectively.

The chemical composition of the samples was determined using inductively coupled plasma optical emission spectrometry (ICP–OES) on a Liberty 200 spectrometer (Agilent Technologies 5100 ICP–OES, Varian, Palo Alto, Santa Clara, CA USA). 20 mg of CaP and CaP–HS were added to 15 mL of a HNO_3_ solutions and placed in an ultrasonic bath sonicator at 37 °C until samples complete dissolution. Solutions were then cooled at room temperature and added with water up to 100 mL before the ICP analysis. Ca, P, and Mg concentration were then measured by their atomic emission at the following wavelengths: 422.673 nm for Ca, 213.618 nm for P and 279.553 nm for Mg.

Specific surface areas of samples were measured through N_2_ gas adsorption by the Brunauer–Emmett–Teller (BET) method using a Surfer instrument (Thermo Fisher Scientific Inc., Waltham, MA, USA). Samples were degassed at 100 °C for 3 h under vacuum before the analysis.

Samples X-Ray Diffraction (XRD) patterns were collected by a DS Advance Diffractometer (Bruker), equipped with a Lynx-eye position sensitive detector, with a CuKα radiation (λ = 1.54178 Å), at 40 kV and 40 mA. The spectra were recorded in the 10–60° 2θ range with a step size of 0.02° and a scanning speed of 0.5 s. Rietveld refinement for phase quantification was performed with the software TOPAS5, and the percentage of each phase in terms of wt.% was refined considering a multiphase system, using tabulated atomic coordinates of hydroxyapatite (ASTM Card file No. 09-0432), and β-TCP (ASTM Card file No. 09-0169).

Equation (1) was used to calculate the crystallinity degree of each sample:

Crystallinity [%] = 100·C/(A + C)(1)

where C was the sum of peaks area and A was the area between the peaks and the background in the diffraction pattern [36].

Scherrer’s formula was used to calculate the size of hydroxyapatite crystallites along the c-axis and along the a/b-axis [37] taking into accounts the diffraction peaks located at 2θ values of 25.8° and 39.7° corresponding to the (002) and (310) reflections, respectively.

### 4.2. Plant Experiment Setup

Seeds of *Diplotaxis tenuifolia* L. and *Valerianella locusta* L. cv Trophy were purchased from TuttoGIARDINO (Udine, Italy). The experiment was carried out under controlled conditions (temperature: within 20 °C–23 °C, PAR: 500 μmol m^−2^ s^−1^, 12 h day^−1^). Respectively, 25 and 20 seeds were soaked in deionized water for 15 min and placed into each 90 mm Petri dishes containing 40 mL of half-strength 0.5%-agar-solidified the Hoagland solution (adjusted to pH = 7.0), which represented the control treatment (i) as well as the substrate for preparing the following treatments: (ii) humic substance (HS, 16 mg L^−1^), (iii) CaP (4000 mg L^−1^), (iv) CaP coated with HS (CaP-HS, 4000 mg kg^−1^). The CaP microparticles were sonicated for 20 min in 20 mL of deionized water before adding to the Hoagland solution for treatments (iii) and (iv); the addition was done when the solution temperature fell between 60 °C and solidification temperature, while stirring.

The duration of the experiment from sowing to harvest was 20 days (Figure S6). Four replicates for each treatment and for each species were prepared for a total of 32 Petri dishes that were kept closed for the whole duration of the experiment. At harvest, germination was calculated as the ratio of germinated seeds out of the total seeds in each Petri dish. Seedlings were carefully removed, washed with deionized water and photographed; photos were processed with the public domain Java image processing software Image J [38] to measure the root lengths that were calculated for each petri dish as the average of the longest root of the plants. For each treatment, half of the plants of each dish were used for the ATP activity while the other half were used for the element content and the biometric parameters (root length, wet and dry weight).

### 4.3. Cellular ATP Activity Determination

For the determination of cellular ATP, shoot portions were weighted (100 ± 20 mg DW) and frozen by liquid nitrogen. A fine powder was obtained by grinding, and it was used for cellular ATP measurement, according to Mattiello et al. [39]. An aliquot (20 ± 2 µL) of shoot soluble fraction was added to the incubation mixture. The ATP calibration curve was performed for each experiment and the sample concentrations were then calculated by interpolation.

### 4.4. Macroelements in Plant Seedlings

To quantify the total content of Ca, K, Mg, and P in roots and shoots of the plant species, seedlings were rinsed with deionized water and were oven-dried at 60 °C for three days. The total biomass of each fraction was digested on a microwave oven (ETHOS EASY digestion system, Milestone, Italy), using 9 mL of HNO_3_ and 1 mL of H_2_O_2_ in Teflon cylinders at 180 °C, following the USEPA test method 3052 (1996). Plant extracts were and filtered with PTFE 0.45 μm membrane syringe filters and diluted prior the ICP–OES (5800, Agilent Technologies Inc., Palo Alto, Santa Clara, CA USA) analysis; scandium was used as internal standard.

### 4.5. Data Analysis

All statistical analyses were performed in R (v. 4.0.3) [40] Effects of HS presence, CaP presence and their interaction on parameters considered were assessed by two-way ANOVA. When necessary, variables were subjected to logarithmic transformation prior to analysis in order to achieve ANOVA assumptions. A posteriori comparison of individual means was performed using Tukey’s test (*p* < 0.05).

## 5. Conclusions

In this work, calcium phosphate particles (CaPs) were extracted from *Salmo salar* bones following a circular economy approach. The obtained materials were then functionalized with humic substances (HS) by a straightforward process. The presence of HS did not alter the bulk physicochemical properties of CaP, with the exception of the net surface charge that became more negative. CaP–HS together with the respective controls were tested against *Diplotaxis tenuifolia* and *Valerianella locusta* seedlings up to 20 days. The results showed that, even though they exhibited a similar tendency to accumulate Ca and P in hypogeal tissues, the two species have different reactions to the treatments in terms of nutrient uptake and translocation. At the foliar level, CaP and CaP–HS were found to significantly increase the accumulation of P in the leaves of *D. tenuifolia*, while the treatment with HS was found to increase only that of Ca in *V. locusta* leaves. Notably, CaP was found to decrease the translocation of Ca in the leaves of *D. tenuifolia* and of both Ca and P in the leaves of *V. locusta*. Finally, the combination of HS with CaP showed promising results in terms of P uptake and translocation only for *D. tenuifolia*, but its biostimulating effect in the early stage growth of plants was low. We hypothesize that this was due to (i) HS low concentration on CaP; (ii) experimental conditions used for seedling growth; and (iii) low response of the tested species to the selected HS. These aspects will be taken into account and investigated more in depth in future works.

## Figures and Tables

**Table 1:** Phase, chemical composition and surface charge of the samples.

Sample	CaP	CaP–HS
HA (wt.%) ^a^	42.8 ± 1.7	42.6 ± 1.3
β-TCP (wt.%)^a^	57.1 ± 1.7	57.4 ± 1.2
CI (a.u.) ^a^	61.4 ± 0.5	61.2 ± 0.5
Ca (wt.%) ^b^	34.0 ± 0.3	33.5 ± 0.6
P (wt.%)^b^	18.7 ± 0.2	18.3 ± 0.4
K (wt.%) ^b^	1.64 ± 0.02	1.11 ± 0.01
Mg (wt.%) ^b^	0.69 ± 0.01	0.67 ± 0.01
Na (wt.%) ^b^	1.80 ± 0.34	1.26 ± 0.03
Ca/P molar ratio ^b^	1.41 ± 0.01	1.41 ± 0.01
Humic acid (wt.%) ^c^	-	0.4
ζ-potential ^d^	−23.0 ± 1.0	−29.5 ± 0.2

^a^ Determined by XRD, ^b^ Determined by ICP-OES, ^c^ Determined by TGA, ^d^ Determined by DLS.

**Table 2:** Seedling root length, root, and shoot dry weight, and ATP concentration in shoots of *Diplotaxis tenuifolia*. Data are expressed as mean ± standard deviation (n = 4). Different letters indicate statistically significant difference between treatments at Tukey’s post-hoc test (*p* ≤ 0.05).

Treatments	Root Length	Roots DW	Shoot ATP	Shoot DW
	(mm plant^−1^)	(mg plant^−1^)	(nmol g^−1^ DW)	(mg plant^−1^)
Ctrl	65.0 ± 8.83	5.08 ± 1.75 B *	0.094 ± 0.026	21.0 ± 0.75 b
HS	49.3 ± 10.4	3.88 ± 1.72 B	0.115 ± 0.031	24.7 ± 2.33 ab
CaP	56.7 ± 7.39	7.63 ± 2.46 A	0.093 ± 0.023	25.0 ± 3.83 a
CaP–HS	56.4 ± 2.33	6.28 ± 2.56 A	0.123 ± 0.031	21.3 ± 2.27 ab

* Capital letters beside the figures mean that the effect of the CaP treatment is significant for the variable in question according to the Tukey’s post-hoc test, while small letters mean that the interaction CaP × HS is significant.

**Table 3:** Seedling root length, root and shoot dry weight, and ATP concentration in shoots of *Valerianella locusta*. Data are expressed as mean ± standard deviation (n = 4). Different letters indicate statistically significant difference between treatments at Tukey’s post-hoc test (*p* ≤ 0.05).

Treatments	Root Length	Roots DW	Shoot ATP	Shoot DW
	(mm plant^−1^)	(mg plant^−1^)	(nmol g^−1^ DW)	(mg plant^−1^)
Ctrl	45.2 ± 4.54 b *	6.23 ± 1.46	0.090 ± 0.011	15.6 ± 1.78 b *
HS	48.2 ± 4.79 a	6.00 ± 0.84	0.087 ± 0.005	18.7 ± 0.05 a
CaP	41.8 ± 4.50 b	6.27 ± 0.77	0.078 ± 0.011	16.1 ± 1.74 b
CaP–HS	54.3 ± 5.05 a	7.73 ± 0.33	0.083 ± 0.006	17.7 ± 0.33 a

* Capital letters beside figures mean that the effect of the CaP treatment is significant for the variable in question according to the Tukey’s post-hoc test, while small letters mean that the interaction CaP × HS is significant.

## Data Availability

The data that support the findings of this study are available from the corresponding author, upon reasonable request.

## Supplementary Materials

The following are available online at, Figure S1: Correlation plot performed on global data set comparing all the considered variables measured for for *Diplotaxis tenuifolia* (A) and *Valerianella locusta* (B)., Figure S2: Concentration of Mg (A) and K (B) in roots of *Diplotaxis tenuifolia*., Figure S3: Concentration of Mg (A) and K (B) in leaves of *Diplotaxis tenuifolia*, Figure S4: Concentration of Mg (A) and K (B) in roots of *Valerianella locusta.*, Figure S5: Concentration of Mg (A) and K (B) in leaves of *Valerianella locusta*., Figure S6: Plantlets of *Diplotaxis tenuifolia* and *Valerianella locusta* in Petri dishes 20 day after sowing., Table S1: Two-way ANOVA applied on morphological and biochemical variables measured on *Diplotaxis tenuifolia*., Table S2: Two-way ANOVA applied on morphological and biochemical variables measured on *Valerianella locusta*., Table S3: Germination percentage, root specific weight, and total seedling dry weight of *Diplotaxis tenuifolia*., Table S4: Germination percentage, root specific weight, and total seedling dry weight of *Valerianella locusta*.

## Sample Availability

Samples of the compounds are available from the authors.
