# Supplementary material for: Calcium Phosphate Particles Coated with Humic Substances: A Potential Plant Biostimulant from Circular Economy
Source: Molecules. 2021 May 10;26(9):2810. doi: 10.3390/molecules26092810 (PMC8126095; doi:10.3390/molecules26092810)
Supplement: Supplementary file 1 [file molecules-26-02810-s001.zip › molecules-1154267-Supplementary.pdf]

molecules

Supplementary materials

Calcium Phosphate Particles Coated with Humic Acids: A Poten-Tial Plant Biostimulant from Circular Economy

Alessio Adamiano [1], Guido Fellet [2], Marco Vuerich [2], Dora Scarpin [2], Francesca Carella [1], Clara Piccirillo [3], Jong-Rok Jeon [4], Alessia Pizzutti [5,2], Luca Marchiol [2], Michele Iafisco[1]

1 Institute of Science and Technology for Ceramics (ISTEC), National Research Council (CNR), Via Granarolo 64, 48018 Faenza (RA), Italy;
alessio.adamiano@istec.cnr.it; francesca.carella@istec.cnr.it; michele.iafisco@istec.cnr.it;
2 Department of AgriFood, Animal and Environmental Sciences, University of Udine, via delle Scienze 206, 33100 Udine, Italy;
guido.fellet@uniud.it; vuerich.marco@spes.uniud.it; scarpin.dora@spes.uniud.it; luca.marchiol@uniud.it
3 Institute of Nanotechnology (NANOTEC), National Research Council (CNR), Campus Ecoteckne, Via Monteroni, 73100 Lecce, Italy; clara.piccirillo@nanaotec.cnr.it;
4 Department of Agricultural Chemistry and Food Science & Technology and IALS, Gyeongsang National University, Jinju 52828, Republic of Korea; jrjeon@gnu.ac.kr;
5 Department of Life Sciences, University of Trieste, Via Licio Giorgieri 10, 34127, Trieste, Italy
alessia.pizzutti@phd.units.it

Table S1. Two-way ANOVA applied on morphological and biochemical variables measured on *Diplotaxis tenuifolia*. Data are mean ± standard deviation (n = 4). Different symbols indicate statistically significance of the analyzed factors (***, p ≤ 0.001; **, p ≤ 0.01; *, p ≤ 0.05).

Parameter	Treatment	Df	F-value	p-value
Roots DW	CaP	1,14	5.5	0.034 *
Shoot DW	HS	1,12	0.00	0.985
	CaP	1,12	0.05	0.832
	CaP x HS	1,12	8.49	0.013 *
log(Ca root)	CaP	1, 14	274	$2{\cdot}10^{-10}$ ***
log(P root)	CaP	1,14	210	$8{\cdot}10^{-10}$ ***
K root	HS	1,12	11.08	0.006 ***
	CaP	1,12	1.16	0.302
	CaP x HS	1,12	6.29	0.027 *
Mg root	HS	1,13	6.44	0.025 *
	CaP	1,13	19.29	$7{\cdot}10^{-4}$ ***
Ca leaf	CaP	1,12	4.96	0.046 *
	HS	1,12	7.11	0.021 *
	CaP x HS	1,12	7.20	0.020 *
P leaf	HS	1,11	28.1	$3{\cdot}10^{-4}$ ***
	CaP	1,11	54.3	$1{\cdot}10^{-5}$ ***
	CaP x HS	1,11	8.5	0.014 *
K leaf	HS	1,12	7.75	0.017 *
	CaP	1,12	0. 22	0.646
	CaP x HS	1,12	14.47	0.002 **
Mg leaf	HS	1,12	0.17	0.689
	CaP	1,12	1.60	0.230
	CaP x HS	1,12	5.51	0.037 *

Table S2. Two-way ANOVA applied on morphological and biochemical variables measured on *Valerianella locusta*. Data are mean ± standard deviation (n = 4). Different symbols indicate statistically significance of the analyzed factors (***, p ≤ 0.001; **, p ≤ 0.01; *, p ≤ 0.05).

Parameter	Treatment	Df	F-value	p-value
Root length	HS	1,14	9.37	0.009 **
Shoot DW	HS	1,14	14.2	0.002 **
log(Ca root)	CaP	1, 13	3487	$2{\cdot}10^{-16}$ ***
log(P root)	HS	1,11	5.29	0.042 *
	CaP	1,11	9299.74	$2{\cdot}10^{-16}$ ***
	CaP x HS	1,11	8.29	0.015 *
K root	HS	1,12	2.57	0.135
	CaP	1,12	0.24	0.631
	CaP x HS	1,12	10.96	0.006 **
Mg root	HS	1, 13	9.96	0.008 **
Ca leaf	HS	1,13	12.5	0.004 **
	CaP	1,13	12.8	0.003 **
P leaf	CaP	1,12	28.8	$2{\cdot}10^{-4}$ ***
	HS	1,12	16.6	0.002 **
	CaP x HS	1,12	18.6	0.001 **
K leaf	CaP	1,13	7.64	0.016 *

Table S3. Germination percentage, root specific weight, and total seedling dry weight of *Diplotaxis tenuifolia*. Data are mean ± standard deviation (n = 4). Different letters indicate statistically significant difference between treatments at Tukey's post-hoc test ($p \leq 0.05$).

Treatments	Germination (%)	Root specific weight (mg mm^{-1})	Total DW (mg $plant^{-1}$)
Ctrl	54 ± 8.33 a	0.077 ± 0.02 a	26.1 ± 2.12 a
HS	58 ± 2.31 a	0.079 ± 0.03 a	28.6 ± 3.99 a
CaP	59 ± 5.03 a	0.135 ± 0.04 a	32.6 ± 5.28 a
CaP-HS	56 ± 10.3 a	0.113 ± 0.05 a	27.5 ± 4.33 a

Table S4. Germination percentage, root specific weight, and total seedling dry weight of *Valerianella locusta*. Data are mean ± standard deviation (n = 4). Different letters indicate statistically significant difference between treatments at Tukey's post-hoc test ($p \leq 0.05$).

Treatments	Germination (%)	Root specific weight (mg mm^{-1})	Total DW (mg $plant^{-1}$)
Ctrl	44 ± 4.79 a	0.140 ± 0.04 a	21.8 ± 2.94 a
HS	53 ± 2.89 a	0.125 ± 0.02 a	24.7 ± 0.84 a
CaP	51 ± 4.78 a	0.152 ± 0.03 a	22.4 ± 2.41 a
CaP-HS	50 ± 7.07 a	0.143 ± 0.02 a	25.4 ± 0.51 a

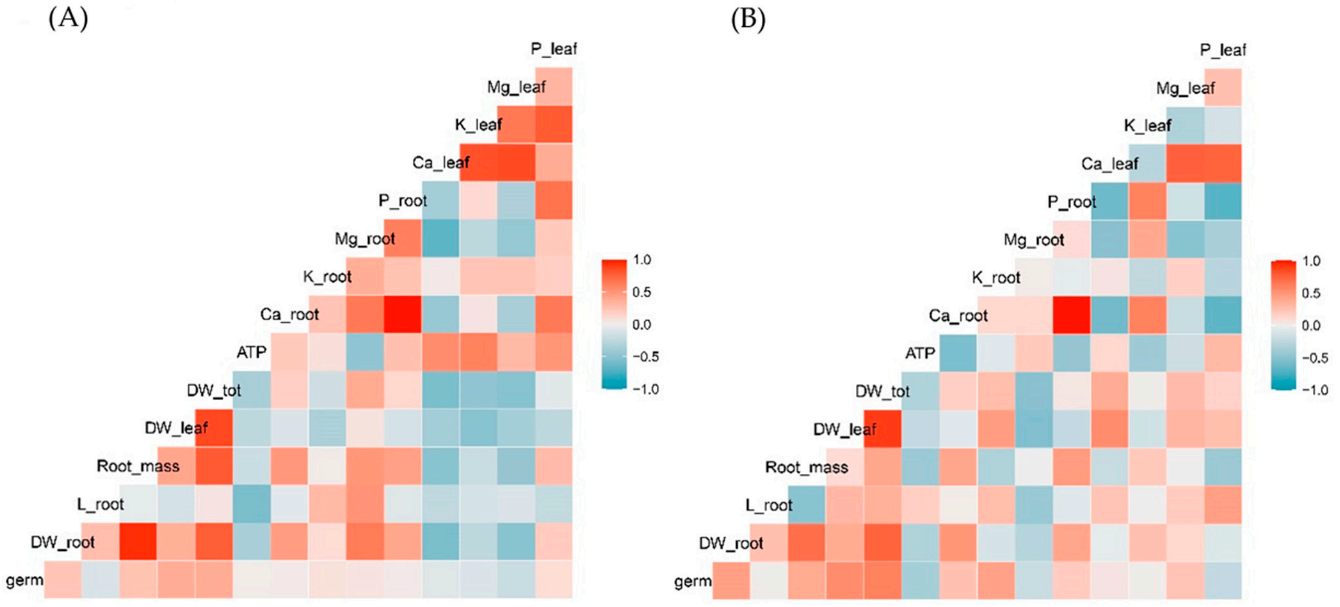

Figure S1. Correlation plot performed on global data set comparing all the considered variables measured for for *Diplotaxis tenuifolia* (A) and *Valerianella locusta* (B). Chromatic palet on the left indicates the correlation degree.

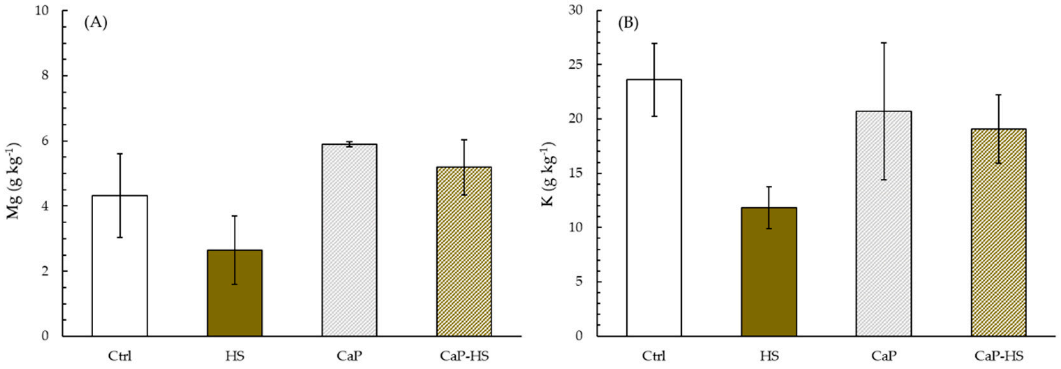

Figure S2. Concentration of Mg (A) and K (B) in roots of *Diplotaxis tenuifolia*. Data are mean ± standard devia-tion (n = 4). When the interaction between experimental factors (CaP x HS) was significant at ANOVA, different letters were used to indicate statistically significant differences between treatments at Tukey's *post-hoc* test ($p \leq 0.05$).

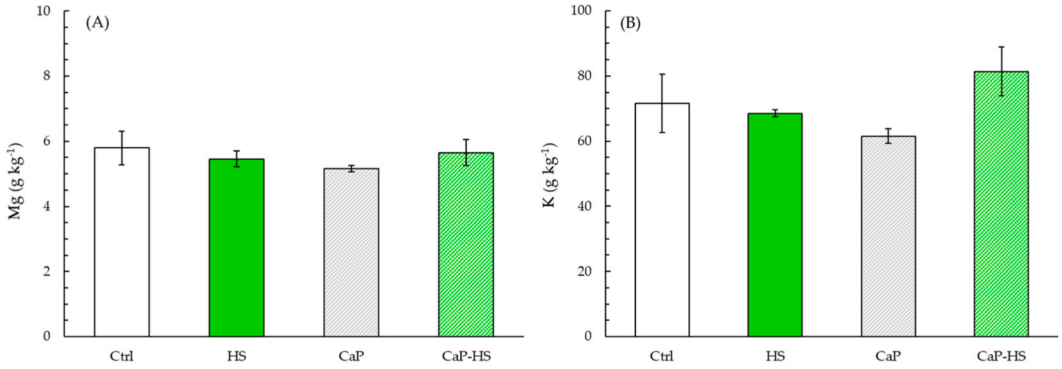

Figure S3. Concentration of Mg (A) and K (B) in leaves of *Diplotaxis tenuifolia*. Data are mean ± standard deviation (n = 4). When the interaction between experimental factors (CaP x HS) was significant at ANOVA, different letters were used to indicate statistically significant differences between treatments at Tukey's *post-hoc* test ($p \leq 0.05$).

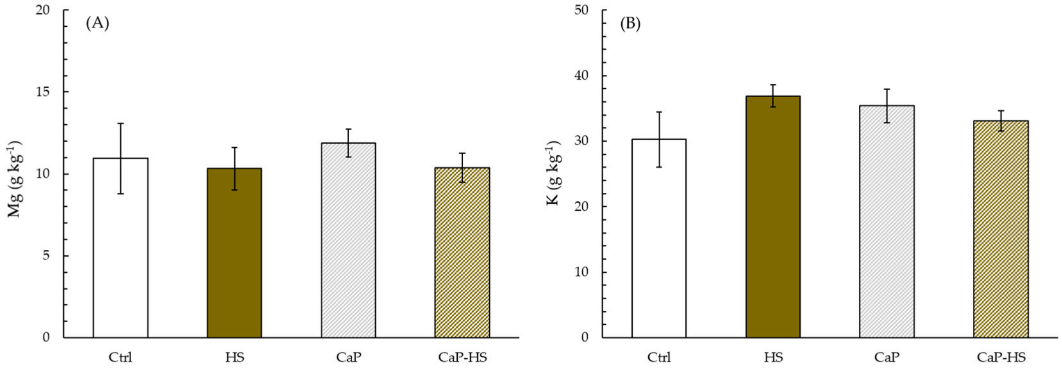

Figure S4. Concentration of Mg (A) and K (B) in roots of *Valerianella locusta*. Data are mean ± standard deviation (n = 4). When the interaction between experimental factors (CaP x HS) was significant at ANOVA, different letters were used to indicate statistically significant differences between treatments at Tukey's *post-hoc* test ($p \leq 0.05$).

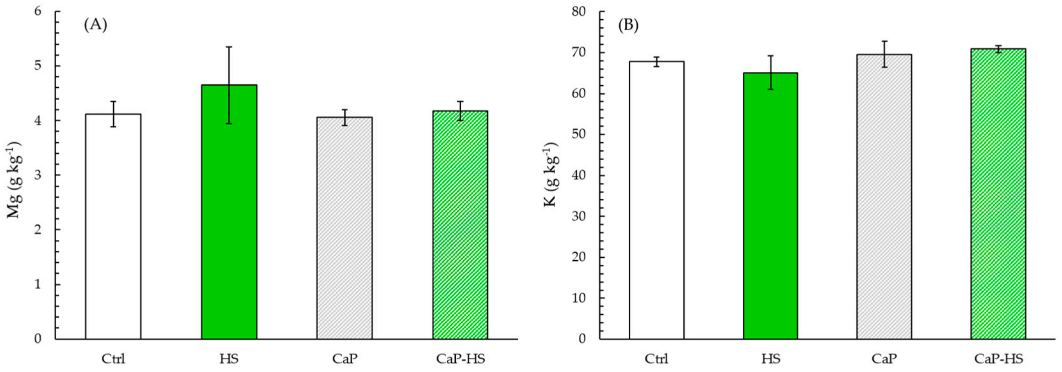

Figure S5. Concentration of Mg (A) and K (B) in leaves of *Valerianella locusta*. Data are mean ± standard deviation (n=4). When the interaction between experimental factors (CaP x HS) was significant at ANOVA, different letters were used to indicate statistically significant differences between treatments at Tukey's *post-hoc* test ($p \leq 0.05$).

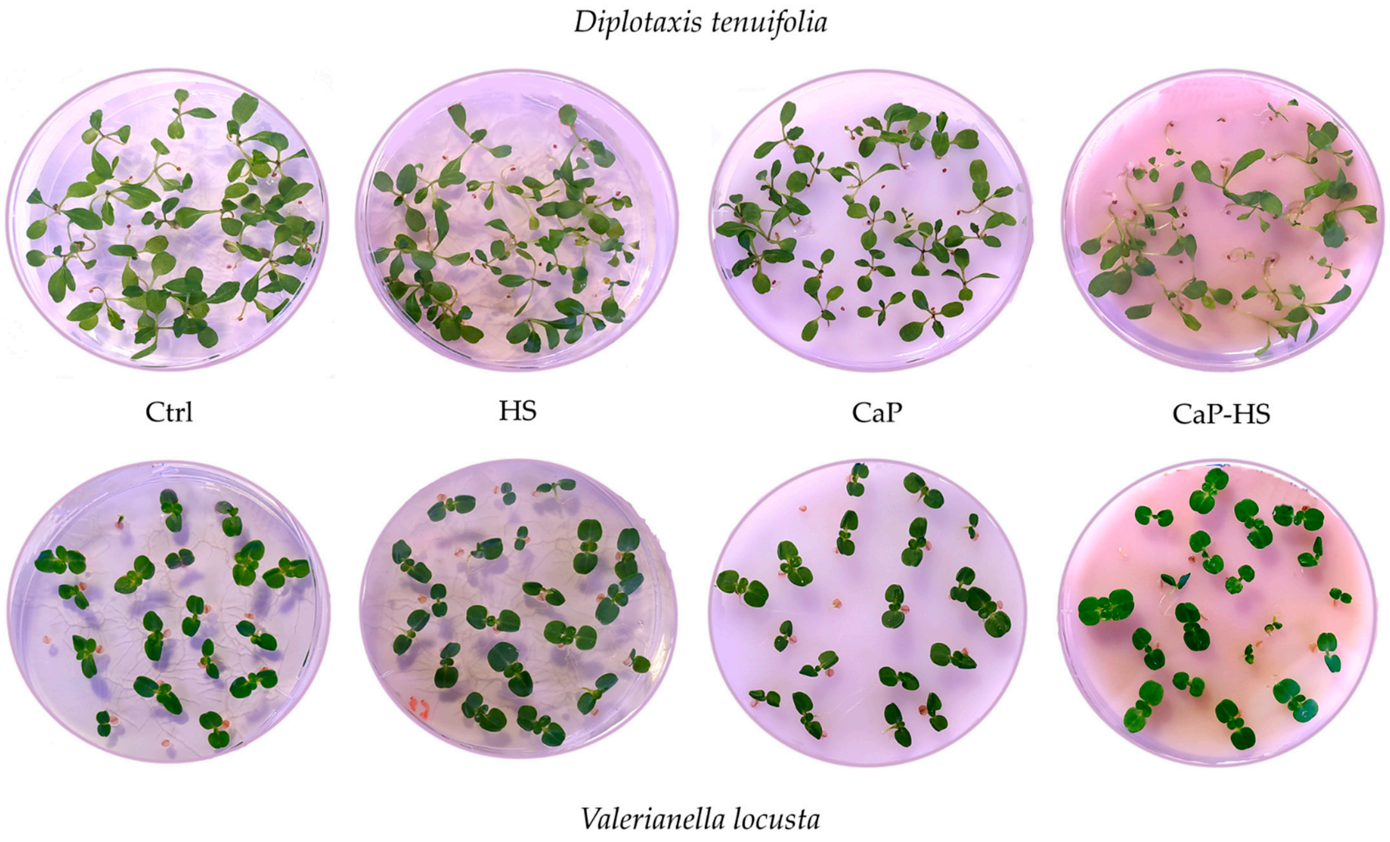

Figure S6 Plantlets of *Diplotaxis tenuifolia* and *Valerianella locusta* in Petri dishes 20 day after sowing.
